# Interaction processes of ciprofloxacin with graphene oxide and reduced graphene oxide in the presence of montmorillonite in simulated gastrointestinal fluids

**DOI:** 10.1038/s41598-017-02620-4

**Published:** 2017-05-31

**Authors:** Shuai Ma, Yang Si, Fei Wang, Lei Su, CongCong Xia, Jun Yao, Huilun Chen, Xingyu Liu

**Affiliations:** 1 School of Energy & Environmental Engineering, University of Science and Technology Beijing, 30 Xueyuan Road, 100083 Beijing, China; 2 Beijing Key Laboratory of Resource-oriented Treatment of Industrial Pollutants, University of Science and Technology Beijing, 30 Xueyuan Road, 100083 Beijing, China; 3 PPSM, CNRS UMR 8531, ENS-Cachan, 61 av President Wilson, 94230 Cachan, France; 4 Research Center for Bioengineering and Sensing Technology, School of Chemistry and Biological Engineering, University of Science and Technology Beijing, 100083 Beijing, China; 5 School of Water Resource and Environmental Engineering, Sino-Hungarian Joint Laboratory of Environmental Science and Health, China University of Geosciences (Beijing), 29 Xueyuan Road, Haidian District, 100083 Beijing China; 6 National Engineering Laboratory of Biohydrometallurgy, General Research Institute for Nonferrous Metals, 100088 Beijing, China

## Abstract

This study investigated the interaction processes of ciprofloxacin (CIP) with graphene oxide (GO) and reduced GO (rGO) in presence of montmorillonite (Mont) in simulated gastrointestinal fluids. The order of CIP adsorption affinity was rGO+Mont > GO+Mont > rGO+Mont+pepsin > rGO > GO+Mont+pepsin > Mont > Mont+pepsin > GO > rGO+pepsin > GO+pepsin in simulated gastric fluid. Mont enhanced the adsorption of CIP on GO and rGO due to hydrated Si species coating on GO and rGO in the simulated gastric fluid. Meanwhile, π–π interaction between CIP and graphene caused the great shift of two cyclopropyl CH_2_ and one cyclopropyl in CIP molecules. And GO, rGO, and Mont interacted mainly with CIP by COOH groups. CIP and pepsin molecules could intercalate and increase the basal spacing of Mont as well. After the various interaction systems of adsorbent-adsorbate transferring to the simulated intestinal fluid, CIP was continuously adsorbed by GO and rGO. In addition, adsorbed CIP was released from Mont into the solution through electrostatic repulsion. The decrease ratio of CIP was the lowest in the GO/rGO+Mont+pepsin systems. Therefore, the mixture of Mont and GO/rGO decreased the CIP concentration in gastrointestinal fluid to weaken further antibiotic activity of CIP.

## Introduction

Antibiotics are extensively used for human and veterinary medicine purposes. Antibiotics such as disease-fighting agents and prophylactics are also widely used in the animal husbandry^1^. 70–90% of antibiotics cannot be metabolized by livestock and remain in the gastrointestinal fluid of livestock^2^. Thus, these antibiotics may interact with other substances in the gastrointestinal fluid. Their interaction processes and adsorption strength might affect the transport behavior of ciprofloxacin (CIP) in the gastrointestinal fluid.

Recently, the graphene industry has dramatically developed rapidly. Graphene production was estimated to be 120 tons per annual (tpa) in 2015, and this value is expected to increase to 1200 tpa in 2019^3^. Therefore, graphene development and industrial application are expected to increase intentional or unintentional human or animal exposure, causing health risk concerns due to their potential ecological effects^4^. Given its hydrophobicity and sp^2^ carbon atom structure, graphene can interact with aromatic organic chemicals through hydrophobic and π–π interactions. Owing to its excellent interaction properties, graphene offers several opportunities and innovative solutions in various applications, such as contaminant removal and drug delivery^5–7^. However, graphene nanomaterials could be released into aquatic environments during the manufacture, use, and disposal of graphene-enabled products^8, 9^. During its life cycle, graphene may enter the gastrointestinal tract of livestock through food or drink consumption. For example, graphene could be partly absorbed and accumulated in aquatic life (e.g., *Daphnia magna*)^10^, which could further enter the gastrointestinal tract through the food chain.

CIP is a second-generation fluoroquinolone antibiotic widely used because of its broad spectrum activity and favorable *in vivo* absorption. Therefore, the adsorption interaction between graphene and CIP would occur in the gastrointestinal fluid. Graphene hydrogels exhibit excellent adsorption capacity (235.6 mg/g) for CIP mainly through π–π electron donor–acceptor (EDA), H-bonding, and hydrophobic interactions^11^. In addition, graphene oxide (GO) adsorbs CIP with a maximum adsorption capacity of 379 mg/g mainly through electrostatic attractions and H-bonding interaction^12^. These findings suggest that the antibiotic activity of CIP decreases after adsorption by GO or reduced graphene oxide (rGO). The strong adsorption of CIP on adsorbents decreases the amount of available CIP for disease treatment. However, the adsorption behavior of graphene and CIP in the gastrointestinal fluid remains poorly understood to date. Furthermore, the interaction mechanisms (e.g., π–π EDA interaction) between graphene and CIP need to be verified because of the lack of evidence on the π–π interaction between CIP and graphene.

Montmorillonite (Mont) is 2:1 layer silicate mineral with one octahedral sheet sandwiched by two tetrahedral sheets^13^. Given its large specific surface area (SA), high cation exchange capacity, and drug-carrying capacity, Mont is widely used as a feed additive to treat and prevent animal intestinal diseases^14^. Mont–CIP composite demonstrates a strong antibacterial activity and is an ideal delivery system for CIP molecules^15^. Thus, Mont should always exist in the gastrointestinal tract to alter the adsorption behavior of graphene and CIP. Wu *et al*. reported that Mont can adsorb both cationic and zwitterion forms of CIP by cation exchange and intercalation^16^. However, Zhao *et al*. found that GO does not interact with negatively charged Mont because of electrostatic repulsion^17^. This result suggests that Mont and GO/rGO compete to adsorb CIP in the gastrointestinal fluid. However, the interaction behavior of CIP with rGO/GO in the presence of Mont remains to be reported so far. Several recent studies have reported the adsorption and desorption behaviors of aflatoxin on smectite^18^, phenanthrene on carbon nanotubes^19^, and bisphenol A and 17 α-ethinylestradiol on marine sediment^20^ in simulated gastrointestinal fluids. These organic contaminants released from adsorbents in simulated digestive fluids further produce bio-toxicity or threaten environment health. By contrast, antibiotics must be released in solution to treat diseases efficiently. However, little is known about the potential behavior of CIP with graphene nanoparticles in the absence or presence of Mont in the digestive system. In addition, pepsin enhances the complexity of the interaction system due to competition with CIP on adsorbents^18^. Moreover, the adsorption strength and interaction mechanisms of CIP must be elucidated to understand the transfer behavior of CIP in the gastrointestinal fluid and thus further assess the antibiotic activity of CIP on the basis of adsorption affinity.

The present study aimed to investigate the adsorption behavior of CIP on GO and rGO in the absence and presence of Mont in simulated gastric fluid, and then subsequent interactions of CIP in varying interaction systems after they transferred to the simulated intestinal fluids. During these interaction processes, we also explored the underlying adsorption mechanism using transmission electron microscopy (TEM)/energy-dispersive X-ray spectroscopy (EDX), Fourier transform infrared spectroscopy (FTIR), X-ray powder diffraction (XRD), proton nuclear magnetic resonance (^1^H NMR), and ultraviolet–visible (UV/vis) absorption spectroscopy. These findings provide new insights into the interaction mechanisms between CIP and graphene materials, and into the transfer behavior of CIP with mixed adsorbates (Mont-GO/rGO) in simulated gastrointestinal fluids.

## Results

### Characterization of GO, rGO and Mont

As presented by SEM images (Fig. S1), rGO showed slight aggregation with loose wrinkles. GO displayed relatively flat and heavy stacked surface. Mont morphologies displayed the particles.

The elemental compositions of GO and rGO determined by EDX and BET SA analyses of the GO, rGO, and Mont are listed in Table S1. The O content of GO (43.41%) was higher than that of rGO, indicating that oxygen breaks up the delocalized π band structure of GO^21^. The XRD pattern shows that Mont contains Si, Na, Al, and Mg (Fig. S2). The SAs of rGO (530 m^2^/g) and GO (329 m^2^/g) were much higher than that of Mont (66 m^2^/g). However, the SAs of GO and rGO were lower than the theoretical value (approx. 2630 m^2^/g)^22^ because of the stacking and aggregation driven by van der Waals forces due to their hydrophobicity.

**Figure 1:**
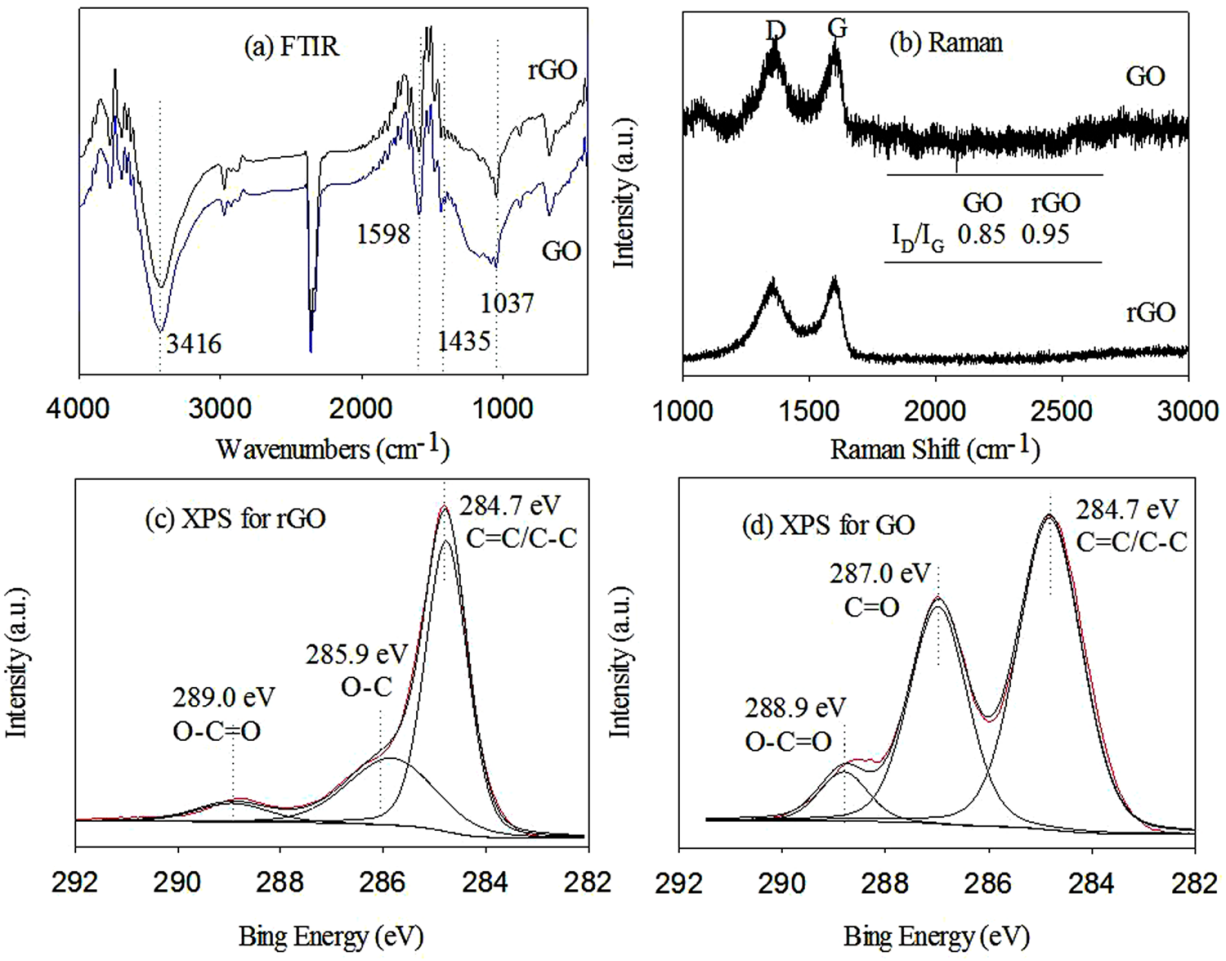
Surface and structural characterization of graphene oxide (GO) and reduced GO (rGO). FTIR spectra of GO and rGO (**a**), Raman spectra of GO and rGO (**b**), XPS spectra of rGO (**c**) and GO (**d**) for C 1 s.

The FTIR spectra of GO and rGO are shown in Fig. 1a. The strong peak at 3416 cm^−1^ was attributed to the stretching vibration of O–H. The peaks at 1598, 1435, and 1037 cm^−1^ were assigned to aromatic C=C, carboxyl O=C–O, and alkoxy C–O, respectively^23^. However, carboxyl O=C–O did not exist for rGO. The abundance of O-containing functional groups was significantly higher on GO than on rGO.

In the Raman spectra (Fig. 1b), GO and rGO exhibited two prominent peaks at 1350 and 1580 cm^−1^, which corresponded to the D and G bands. The D band associates with structural defects and disorders of the sp^2^ carbon domains, whereas, the G band is related to the stretching vibration of sp^2^ carbon atoms^24^. The intensity ratio of the D band to the G band (I_D_/I_G_) of GO (0.85) was lower than that of rGO (0.95), suggesting that the newly formed sp^2^ domains after reduction were smaller but more prevalent^25^. rGO reduced aromatic character, whereas GO can be described as a nonaromatic surface with sp^3^ carbon atoms (bonded with oxygen groups) with isolated aromatic areas and sp^2^ domains^26^. These characteristics differentiate the adsorption behavior of rGO and GO.

The XPS spectra were analyzed to determine the surface functional groups of GO and rGO (Fig. 1c and d, and Fig. S3). Both GO and rGO had significant peaks at ~284.8 and ~289 eV, which were assigned to the sp^2^-hybridized carbons in C=C/C–C and O–C=O, respectively^27^. The differences were observed at 285.9 eV (O–C) for rGO and 287 eV (C=O) for GO. Meanwhile, the two O 1 s spectral peaks at ~533.4 and ~532.08 eV of rGO were attributed to –C–O/–OH and C(O)O/–C=O groups, respectively^23, 28^. However, only GO showed the C(O)O/−C=O peak. In addition, the O/C atomic ratio decreased from 0.43 (GO) to 0.15 (rGO) after the reduction process. These results indicated that the C=O group was partly reduced to the C–O group that the amount of the former group decreased. Therefore, rGO had a relatively high hydrophobicity.

### Adsorption isotherms for CIP on GO, rGO, and Mont in simulated gastric fluid and for pepsin on GO, rGO and Mont at pH 2

**Figure 2:**
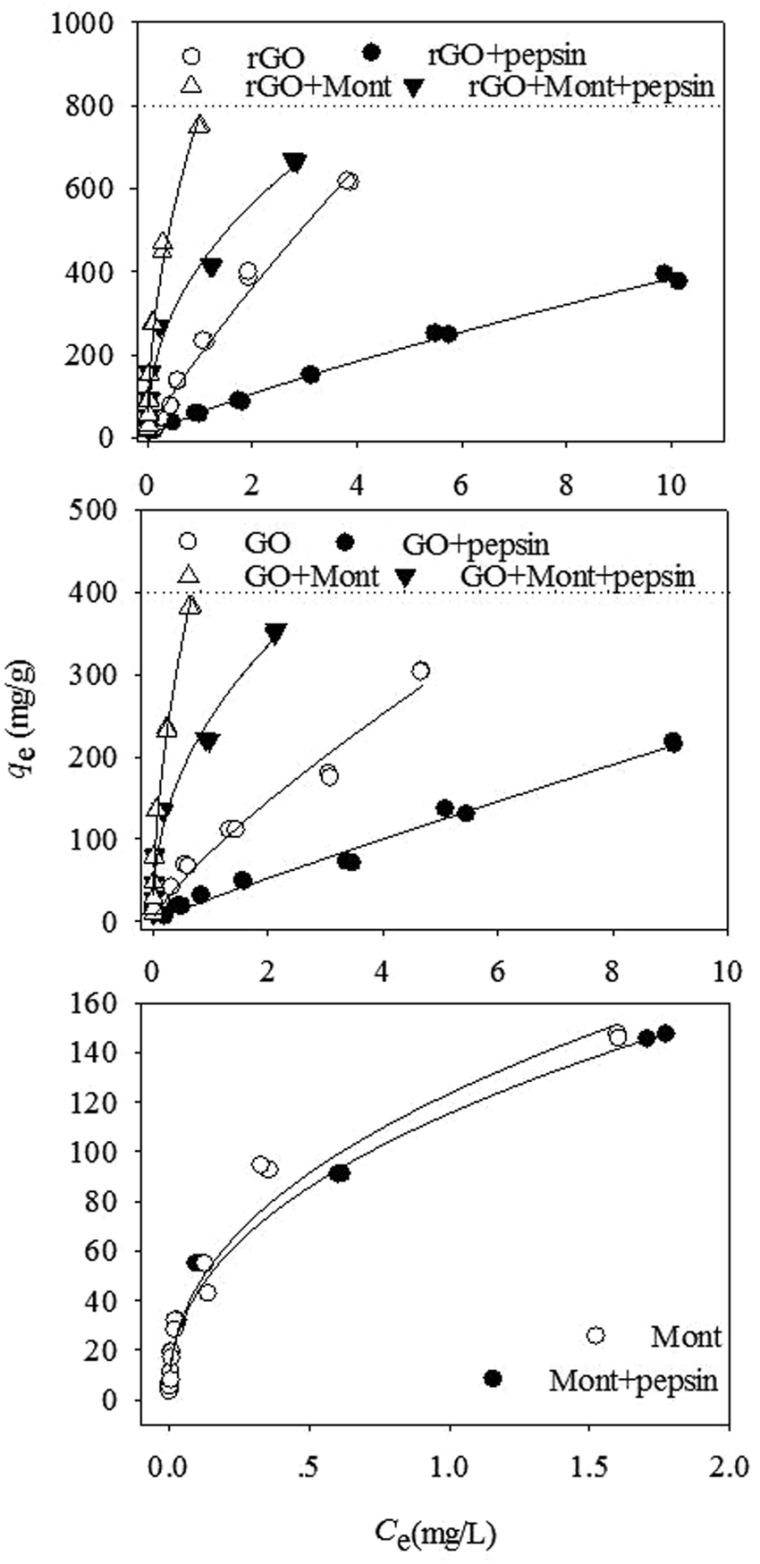
Adsorption of CIP on GO, rGO and Mont under the varying interaction system at pH 2. The solid line is Freundlich model.

The adsorption isotherms of CIP on GO, rGO, and Mont in simulated gastric fluid were fitted using the Freundlich model (Fig. 2), and the regression parameters are listed in Table S2. Meanwhile, the adsorption isotherms of CIP on Mont in the presence/absence of pepsin were also obtained to determine the effect of Mont on adsorption in gastric fluid. Figure 2 shows that all of the isotherms were nonlinear and that the Freundlich *n* values varied under the different interaction systems, indicating the heterogeneity of the surface^29^. The addition of Mont decreased the Freundlich *n* values, indicating that Mont enhanced the heterogeneity of mixed adsorbents (i.e., rGO/GO+Mont). The single point adsorption coefficient (*K*_d_) at *C*_e_ = 2 mg/L (CIP) was calculated on the basis of the Freundlich fitting results (Table S2). The order of CIP adsorption affinity represented by *K*_d_ was rGO+Mont > GO+Mont > rGO+Mont+pepsin > rGO > GO+Mont+pepsin > Mont > Mont+pepsin > GO > rGO+pepsin > GO+pepsin.

Figure 2 shows the adsorption affinities of the single interaction systems (rGO, GO, and Mont). rGO possessed a stronger adsorption affinity than GO and Mont, which contributed to the larger SA of rGO (530 m^2^/g) than GO (329 m^2^/g) and Mont (66 m^2^/g). The SAs of the adsorbents always acted as a main factor influencing adsorption capacity^30^. In addition, the *K*_d_ of Mont (82.9) was slightly greater than that of GO (72.7), which could reflect their different adsorption mechanisms except for the role of SA.

Pepsin is the major enzyme in gastric fluid. As shown in Fig. 2 and Table S2, the presence of pepsin reduced the adsorption of CIP on GO, rGO, and Mont to some extent. The decrease degree of CIP adsorption was the largest for rGO and reached up to 3.4 times. Pepsin slightly affected the adsorption of CIP on Mont (reducing 1.07 times). The effect was negligible at a low concentration of CIP with exposure to Mont, and their isotherms almost overlapped. The decreased adsorption of CIP might be caused by the competition adsorption of CIP and pepsin in gastric fluid. The adsorption isotherms of pepsin on GO, rGO and Mont were obtained to interpret the adsorption strength of pepsin (Fig. S5 and Table S3). The order of pepsin adsorption affinity (*K*_d_) was rGO > GO > Mont, which coincided with the decrease ratios of CIP adsorption. This result indicates that the adsorbed amount of pepsin decided on the decrease degree of CIP adsorption.

**Figure 3:**
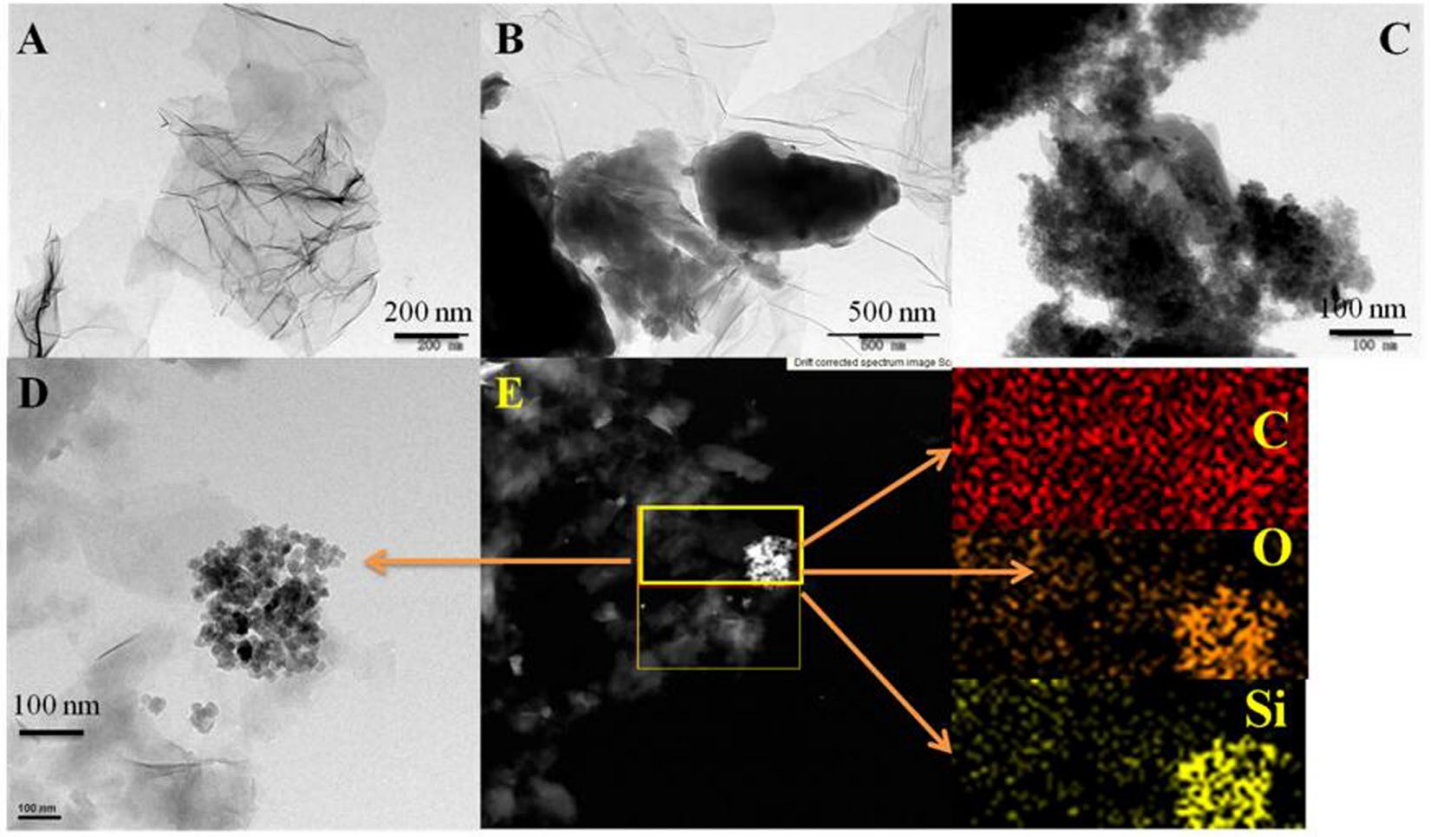
TEM images of rGO+CIP (**A**), rGO+Mont+CIP (**B**), rGO+Mont+pepsin+CIP (**C**), GO+Mont+CIP (**D** and **E**) after adsorption.

However, the mixed adsorbents of GO/rGO+Mont considerably increased CIP adsorption in the absence of pepsin as opposed to the decrease adsorption of CIP on GO, rGO, and Mont in the presence of pepsin. The *K*_d_ values reached up to 552 for rGO+Mont and 351 for GO+Mont. Furthermore, the increase degree of CIP adsorption was even greater than that of the addition of individual rGO/GO and Mont adsorbents. This result may be attributed to other interaction mechanisms aside from the aforementioned π–π EDA, hydrogen bonding, hydrophobic and electrostatic interaction for GO/rGO and cation exchange for Mont. In the mixed adsorbents, GO and rGO should have no interaction with Mont because of the electrostatic repulsion from their positively charged surface at pH 2 (Fig. S4). However, graphene could cover Mont to some extent, or the aggregate of Mont occupied part of the graphene surface (Fig. 3A and B and Fig. S6). This mixture reduced some surface sites of GO, rGO, or Mont to be accessible to CIP. To explain further the increasing adsorption of CIP, we also considered the possible effect of aqueous Si and Al released from the Mont on adsorption of CIP on the mixed adsorbents^31^. The concentrations of Si and Al in solution are shown in Table S4, and the concentration of Si was much greater than that of Al. In addition, Fig. 3D and E show that C, O, and Si were obviously distributed on the surface of mixed GO and Mont in different colors. The released Si was adsorbed on the GO surface. Al was not detected because of its lower than the detection limit.

The addition of pepsin in the GO/rGO+Mont interaction systems decreased CIP adsorption to some extent because of the aforementioned competition adsorption between CIP and pepsin. Pepsin was clearly observed to coat on rGO and Mont surface (Fig. 3C). Meanwhile, the dissolved Al and Si decreased in the presence of pepsin than that in the absence of pepsin. Therefore, pepsin not only suppressed the adsorption of CIP but also reduced the aqueous Al and Si by coating on Mont in gastric fluid.

Compared with the CIP adsorption at pH 2 in simulated gastric fluid, CIP adsorption isotherms were obtained at pH 6.5 (Fig. S7). On the basis of the *K*_d_ values, the adsorption affinity followed the order of rGO+Mont > GO+Mont > rGO > GO > Mont (Table S5). This order was consistent with the order at pH 2. However, the adsorption affinities of CIP were lower at pH 6.5 than at pH 2 because the interaction strength was weakened.

### ^1^H NMR, UV-vis absorption, FTIR, and XRD analyses on the interaction mechanisms between CIP and GO, rGO, and Mont

**Figure 4:**
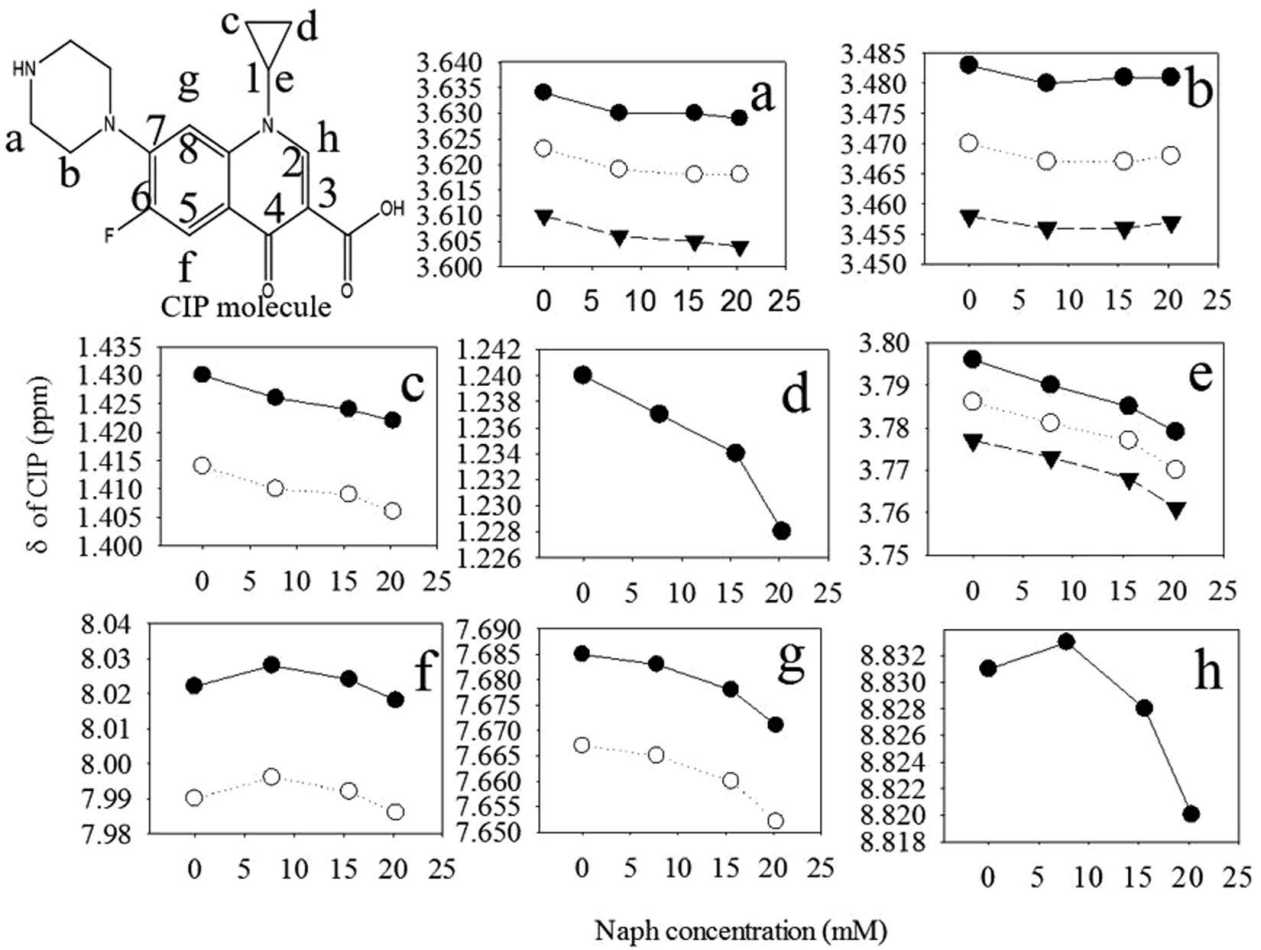
^1^H NMR chemical shift of CIP as π-acceptors in methanol-*d*_4_ affected by complexation with Naph as π-donors. CIP concentration was 7.8 mM, and Naph concentration was 0, 7.8, 15.6 and 20.3 mM. (**a–h**) were noted as H position in CIP molecular.

The π–π EDA complexation between π-electron acceptor and PAHs in solution was supported using ^1^H NMR upfield chemical shifts^32, 33^. The ^1^H NMR spectra of CIP and the CIP–Naph complex were measured (Fig. S8), and detected peaks of CIP were also marked^34, 35^. The chemical shifts (ppm) of proton NMR for the detected peaks were obtained (Figs 4 and S9). The proton on the different positions of CIP molecules in CIP-Naph solution shifted to a different extent in comparison with CIP. Two cyclopropyl CH_2_ and one cyclopropyl CH showed the greatest shifts, their shift ratio reached approximately 0.6%, 1.2%, and 0.5%, respectively. Another proton shift ratio showed the order of proton on C8 (g position) > piperazine H (a position) > proton on C2 (h position) > piperazine H (b position) > proton on C5 (f position).

Solution phase π–π EDA complexes often show a charge-transfer band in the UV/vis region. The presence of π–π charge-transfer absorbance bands of CIP and Naph mixtures in different pH values was determined (Fig. S10). The intensity of the charge-transfer band gradually decreased with increasing solution pH.

**Figure 5:**
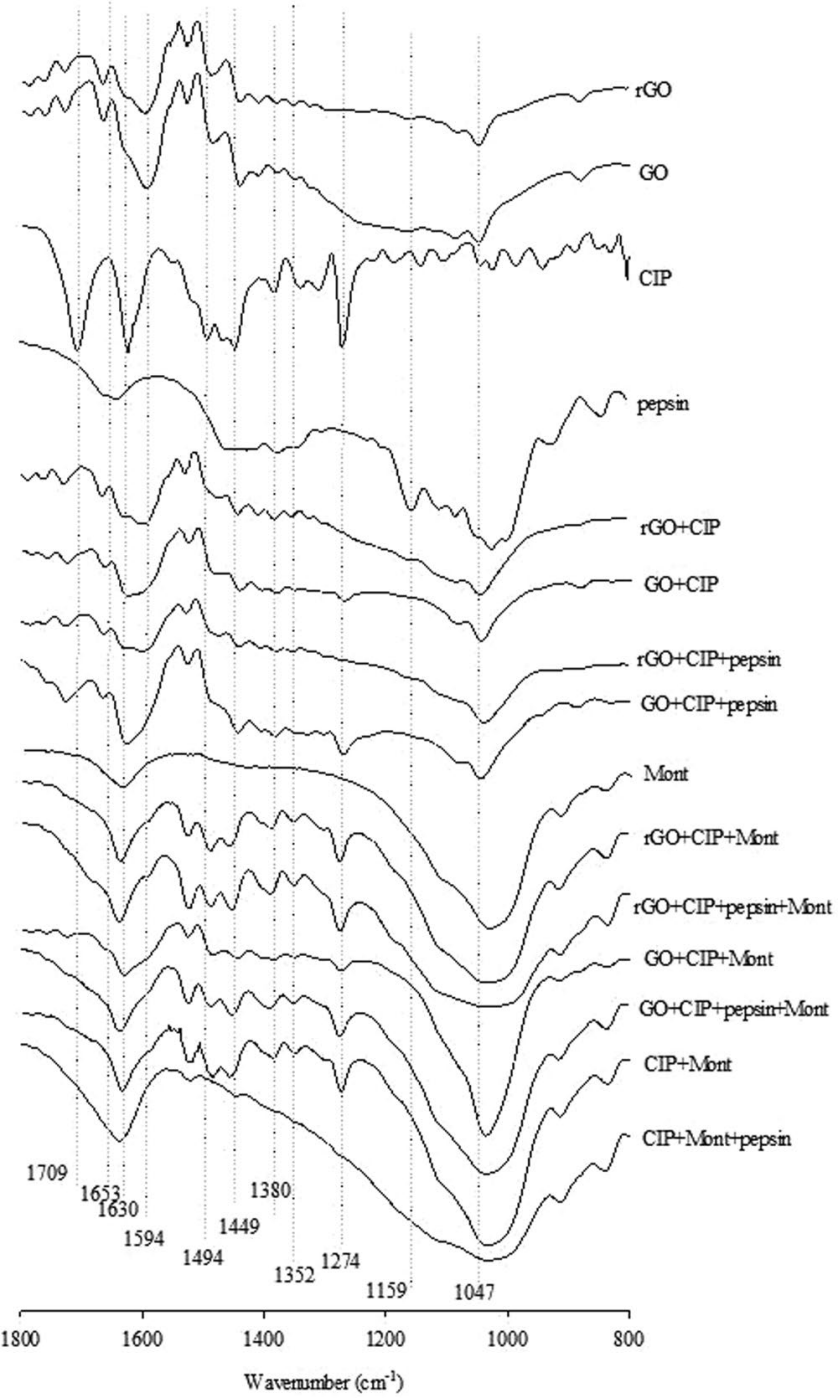
FTIR spectra of GO, rGO and Mont under the varying interaction systems after adsorption at the range of 800 to1800 cm^−1^ at pH 2.

To probe the molecular interaction of CIP with GO, rGO, and Mont in the varying interaction systems, the FTIR spectra of GO, rGO, and Mont after CIP adsorption are shown in Fig. 5 (Fig. S11). The peaks at 1709, 1630, 1494, 1449, 1380, and 1274 cm^−1^ in the CIP spectra were assigned as the carboxylic acid C=O stretch (COOH), ketone C=O stretch, C–N stretch, protonation of amine group in piperazinyl moiety, and C–C stretch, respectively^34, 36^. The 1653 cm^−1^ vibration of pepsin was attributed to amine I^18^. The broad band at 1040 cm^−1^ was attributed to Si–O stretch of di and trioctahedral smectites^36^. Meanwhile, the band at 1709 cm^−1^ obviously disappeared for GO, rGO, and Mont and their mixtures loaded with CIP, indicating that COOH was involved in the interactions.

XRD patterns confirmed the presence of CIP or pepsin in the interlayer space of Mont. The basal spacing of Mont was 12.0 Å, which increased to 13.1, 14.3, and 14.6 Å after adsorption with CIP, pepsin, and CIP-pepsin, respectively (Fig. S12). However, the magnitude of increase was much smaller than the sizes of CIP (13.5 × 3 × 7.4 Å) and pepsin (diameter of 44 Å). After the desorption of CIP and pepsin in simulated intestinal fluids, the *d*_001_-spacing of Mont was reduced to 12.3, 13.3, and 14.4 Å in the presence of CIP, pepsin and CIP-pepsin, respectively.

### Continuous interaction of CIP with adsorbents in simulated intestinal fluid

**Figure 6:**
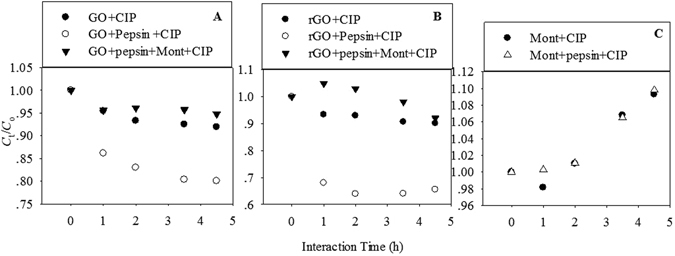
Interaction of CIP with GO, rGO and Mont under the varying interaction systems at the simulated intestinal fluids. *C*_0_ was the concentration of CIP at 0 h, and *C*_t_ was the concentration of CIP at 1, 2, 3.5, and 4.5 h.

Continuous interaction of CIP occurred in the GO, rGO, Mont, and GO/rGO+Mont interaction systems in the presence and absence of pepsin in simulated intestinal fluid (Fig. 6). The desorption kinetics of CIP for the three adsorbents changed differently with interaction time. Desorption curves of CIP in the interaction system containing GO or rGO (Fig. 6A and B) decreased. Obviously, the decrease ratio of CIP concentration followed the order of GO/rGO+Mont+pepsin > GO/rGO > GO/rGO+pepsin, which coincided with the adsorption order of CIP in simulated gastric fluid. For the systems containing Mont (Fig. 6C), the desorption curves increased, indicating that adsorbed CIP was released into the solution. Pepsin exerted no effect on desorption of CIP from Mont.

CIP species became CIP^±^ in simulated intestinal fluid. Meanwhile, GO, rGO, and Mont showed negative charges (Fig. S4), and Mont had more negative charges than GO and rGO. Thus, the electrostatic repulsion between CIP and Mont should be stronger than that between CIP and GO/rGO.

The GO/rGO+Mont+pepsin systems exhibited the lowest decrease ratio of CIP because of the existence of Mont, indicating that the amount of released CIP from Mont was not enough to make the GO/rGO adsorption saturated to some extent. The released CIP from Mont would be adsorbed on GO and rGO.

## Discussions

Our results show the various adsorption behavior of CIP on rGO and GO in the presence of Mont and pepsin in the stimulated gastric fluid. For the single interaction systems containing only GO, rGO and Mont, CIP was adsorbed on Mont via cation exchange between the protonated heterocyclic N atom of CIP and permanent charge sites on Mont^37^. However, CIP interacted with graphene nanomaterials via π–π EDA, hydrogen bonding, and hydrophobic interactions^11^. The zeta potentials of GO, rGO, and Mont were determined as a function of solution pH (Fig. S4). These adsorbents were positively charged at pH 2, but the charges were very low, i.e., 2.82 ± 0.03 mV for rGO, 0.84 ± 1.38 mV for GO, and 2.31 ± 1.79 mV for Mont. In addition, CIP existed in cationic form CIP^+^ because of the protonation of the secondary amine on the piperazine groups when the solution pH was below 6.1 (pK_a1_ = 6.1)^38^. Thus, electrostatic repulsion occurred between positively charged adsorbents and CIP^+^ in simulated gastric fluid. However, the electrostatic repulsion should be weak because of the low charges on GO, rGO, and Mont. As for GO and rGO as characterized by Fig. 1, the high oxygen content on GO could decrease the CIP adsorption through reducing the π–π interactions by localizing the π electrons and depleting the available SA by forming water clusters^39^.

As with the presence of pepsin, CIP adsorption on GO, rGO, and Mont reduced due to the competition adsorption of CIP and pepsin in gastric fluid. Pepsin is a globular protein with a diameter of 44 Å in water^18^, and its conformation extended to B-type after adsorption, whereas CIP has a molecular size of 13.5 × 3 × 7.4 Å^40^. Therefore, pepsin occupied larger adsorption sites than CIP to decrease further the CIP adsorption on the adsorbents. A similar phenomenon was also reported for the decrease adsorption of other organic chemicals (phenanthrene, bisphenol A, and 17α-ethinylestradiol) in the presence of pepsin in simulated gastric fluid^19, 20^. Hence, the decrease adsorption of CIP on the GO, rGO, and Mont existing in gastric fluid induced animals to uptake free CIP.

CIP adsorption on mixed adsorbents of GO/rGO+Mont was enhanced in the absence of pepsin. The dissolved Si and Al could further be adsorbed on rGO and GO. At a low pH of 2, the Al species is Al^3+^, which exhibited the electrostatic repulsion with the positively charged rGO. Hence, Al^3+^ ions were barely adsorbed on rGO or GO at the low pH^41^, and the released Al exerted no obvious effect on the increasing adsorption of CIP in gastric fluid. By contrast, Si was widely distributed on the surface of GO. The hydrated Si was on SiO(OH)_3_^−^ and SiO_2_(OH)_2_^2−^ ^42^, which interacted with CIP through electrostatic attraction to enhance further the adsorption of CIP.

Compared with the adsorption of CIP at pH 2, the interaction strength of CIP was different at pH 6.5. The protonated amino groups in CIP could cause stronger cation-π bonding with π-electrons on the graphene surfaces under the lower pH values^40, 43^. The π–π EDA interaction is discussed in the following part. CIP existed in a zwitterion form (CIP^±^) at pH 6.5. Li *et al*. reported that the electrostatic interaction might be weakened at pH 6.1–8.7, because hydrophobic interaction is enhanced and dominant because of the higher hydrophobicity of CIP^±^^44^. Therefore the adsorption differences may be induced by changing the interaction strength of CIP and rGO, GO, and Mont, where the interaction mechanisms were controlled by environmental conditions.

^1^H NMR upfield chemical shifts of CIP molecules. This result proves that the benzene rings with N-heteroaromatic ring and fluorine group on CIP molecules show a strong electron withdrawing ability as a π-electron acceptor because of N and F. In addition, the magnitudes of upfield shifts increased with the π-donor concentration (Naph). This result suggests the formation of a π–π EDA complex in the solution as reported in a previous study^45^. The charge-transfer band in the UV/vis region further verifies that the protonated amino groups in CIP could strengthen the π-electron withdrawing ability under a low pH.

FTIR spectra showed that COOH was involved in the interactions. This finding coincided with a previous study in which the disappearance of the band was attributed to the formation of a surface complex between Al cation and CIP by the involvement of a carboxylic group^38^. The ketone C=O was not involved because of high proximity with the O–H deformation band of water or the materials^36, 46^. In the presence of Mont, a peak at 1040 cm^−1^ existed in the GO/rGO+Mont and GO/rGO+Mont+pepsin interaction systems. In addition, the peak at 1380 cm^−1^ of CIP did not shift in GO/rGO, and GO/rGO+pepsin but shifted in the presence of Mont. This result suggests that electrostatic interaction occurred between the protonated amine groups and the negative surface charges of Mont.

Increase of basal spacing of Mont determined by XRD indicated that functional groups of CIP and pepsin were only partly inserted into the Mont interlayers^47^. Meanwhile, the greater expansion of the pepsin and pepsin-CIP samples might be due to the larger protein molecules present in the interlayer than in the CIP sample. After desorption of CIP and pepsin in simulated intestinal fluids, the decrease of *d*_001_-spacing of Mont suggested that CIP or pepsin was partly desorbed from the Mont surface. The *d*_001_-spacing of Mont almost no changed in the CIP–pepsin system. The simulated intestinal fluids contained 1000 mg/L of bile salts. However, Wu *et al*., reported that Na^+^ exerts no effect on the desorption of CIP from Mont^38^. Thus, desorption of CIP might result from the other mechanisms because pH 2 increased to 7.5.

Desorption curves of CIP in the interaction system containing GO or rGO (Fig. 6A and B) decreased, suggesting that CIP was adsorbed continuously. Fang *et al*. reported that preferential adsorption at the high-energy sites of nanoparticles complicates sequential desorption at low phenanthrene concentrations^48^. Conversely, the limited high energy sites became saturated and phenanthrene adsorption occurred at low energy sites and high phenanthrene concentrations, which consequently simplified the overall desorption process^48^. In this case, the adsorption of CIP on GO and rGO was not saturated in gastric fluid because of the low concentration of CIP (7 mg/L). Once the interaction systems were transferred into the large volume of intestinal fluid, the free CIP was adsorbed again to reach the adsorption equilibrium.

In simulated intestinal fluid, the CIP adsorbed on Mont was released into the solution again, which increased the CIP concentration in simulated intestinal fluid. By contrast, π-π EDA and hydrophobic interaction still worked^36^. Thus, the adsorption of CIP was continuous, even though these processes were slow. In addition, an increase in pH yielded a lower protonation of the surface of Mont, less OH groups, and less sites for adsorption and H bonding^49^. This phenomenon also resulted in the CIP desorption on Mont. The *d*_001_-spacing of Mont decrease also supported desorption of CIP.

For GO/rGO+Mont+pepsin systems, decrease ratio of CIP was the lowest, indicating that released CIP was adsorbed on GO and rGO again. Therefore, GO and rGO decreased the CIP concentration in the gastrointestinal fluid to further weaken the antibiotic activity of CIP. This phenomenon can promote the application of antibiotics as disease-fighting agents and prophylactics. Hence, a large number of CIP applications would lead to serious environmental risks, such as developing the antibiotic resistance in aquaculture^50^.

The excessive input of GO/rGO-Mont-CIP composites into the environment would result in the further release of CIP. More CIP was available in the gastrointestinal fluid to achieve the antibiotic affect^15^. The active form of CIP results in the development of resistant microbes. In addition, graphene nanoparticles might co-exist with Mont or other minerals in the presence of organic matters^51^, and their interaction would alter the environmental fate of organic chemicals. Therefore, these results benefit the research on adsorption behavior of organic compounds in combined environments. These results may expand to explore the interaction processes of various drugs with two-dimensional transition metal dichalcogenides (2D TDCs) and two-dimensional transition metal oxides (2D TMOs) owing to their widely application, such as drug delivery, therapeutics, biosensing, etc.^52, 53^. Meanwhile TDCs and TMOs have varying layers, like Mont, and hold large surface area, stability in aqueous environments. Hence, TDCs and TMOs might exist, liking graphene and Mont, and interacts with drugs in gastrointestinal fluid. Therefore, TDCs and TMOs should be considered the interaction processes with drug molecule when they are applied in biological systems.

## Materials and Methods

### Materials

CIP, pepsin, and bile salts were purchased from Sigma–Aldrich (St. Louis., MO, USA). The molecular weight, water solubility, *n*-octanol-water partition coefficient (log *K*_ow_), p*K*_a,1_ and p*K*_a,2_ of CIP were 331.33 g/mol, 30 g/L, 0.40, 5.90, and 8.89, respectively^37^. Pepsin (activity, 800–2500 units/mg protein) with a molecular weight of 35 kDa was obtained from porcine gastric mucosa. Bile salts were mixed using 50% sodium cholate (NaC) and 50% sodium deoxycholate (NaDC). Mont (purity > 99.0%) was purchased from Zhenjing Sanding Technology Company (Zhejing, China). rGO and GO were purchased from Chengdu Organic Chemistry Co., Chinese Academy of Sciences. The physicochemical properties of rGO and GO are detailed in Table S1 in the Supplementary Materials.

### Characterization of GO, rGO, and Mont

The properties of GO and rGO were characterized by BET-N_2_ specific SA analysis, Raman spectroscopy, and TEM. The surface functional groups were observed by X-Ray photoelectron spectrometry (XPS) and FTIR. The properties of Mont were measured by BET-N_2_ specific SA analysis, scanning electron microscopy (SEM), and XRD. The XRD patterns matched the montmorillonite reference pattern from the International Centre for Diffraction Data. Detailed information is presented in the Supplementary Information.

### Gastrointestinal Fluids Preparation

Gastrointestinal fluids were simulated and modified as previously described^19^. In brief, the simulated gastric fluid was a NaCl–HCl solution with 0.1 mol/L NaCl (pH 2.0) and 800 mg/L pepsin. Pepsin was not added to a control-simulated gastric fluid to test the effect of pepsin on adsorption. The simulated intestinal fluid was a neutral buffered solution with 0.12 mol/L NaCl, 0.02 mol/L Na_2_CO_3_ (pH 7.5), and 1000 mg/L bile salts. A control experiment (0.01 mol/L CaCl_2_, pH 7) was designed to compare the adsorption behavior of the gastrointestinal fluids. NaN_3_ (200 mg/L) was used in all solutions to minimize bioactivity.

### Adsorption Experiments

Adsorption isotherms were performed using a batch equilibrium method in 40 mL glass vials with Telflon-lined screw caps at 37 °C. rGO (1 mg), GO (2 mg), and Mont (5 mg) were mixed with simulated gastric fluid or background solution of varying concentrations from 0.5 to 20 mg/L, and then shaken at 100 rpm for 4 days. After equilibration, the mixtures were filtered through a 0.2 μm PTFE filter. CIP concentration was quantified using a high-performance liquid chromatography system (Shimadzu LC20AD, Japan) equipped with a fluorescence detector at an Ex/Em wavelength of 280/445 nm. The mobile phase was 20:80 (v:v) of acetonitrile and 0.05 M phosphoric acid, and the flow rate was 1 mL/min^40, 54^. A reversed-phase C18 column (5 μm, 4.6 mm × 150 mm) was used. Adsorbed CIP was calculated directly by the mass difference between the initial and equilibrium concentrations.

For pepsin adsorption, pepsin was dissolved in NaCl-HCl solution to obtain solutions with various concentrations (20–800 mg/L). rGO (1 mg), GO (2 mg), and Mont (5 mg) were added to each vial containing 40 mL of pepsin solution. All vials were shaken for 4 days at 37 °C to reach adsorption equilibrium. After filtering, pepsin concentration was determined with a high-temperature total organic carbon analyzer (Shimadzu TOC-V, Japan) using the nonpurgeable organic carbon method. Adsorbed pepsin was calculated directly by the mass difference between the initial and equilibrium concentrations.

### Microstructure Observation, FTIR Spectroscopy, and XRD Analysis after Adsorption

TEM and SEM were used to observe the morphology of rGO under varying interaction systems, such as rGO+CIP, rGO+CIP+Mont, and rGO+CIP+Mont+pepsin. The disperse status of C, O, and Si in the GO+CIP+Mont interaction system was determined through EDX spectroscopy, and their morphology was obtained using field-emission transmission electron microscopy. Meanwhile, the Al and Si in solution were quantified by inductively coupled plasma atomic emission spectroscopy. Samples for TEM observation were prepared by dropping the homogeneous suspension on a TEM grid made of Cu. Samples for SEM and FTIR analyses were prepared by centrifuging and rinsing three times after adsorption and then freeze-drying.

In order to obtain the enough samples for XRD detection, Mont (30 mg) was used to design a series of experiment systems, including Mont+CIP, Mont+pepsin, and Mont+pepsin+CIP, with the 20 mg/L of CIP and 800 mg/L pepsin at pH 2. Duplicated samples were prepared in simulated gastric fluid. After adsorption equilibrium, one sample was used for CIP desorption in simulated intestinal fluid. The samples were freeze-dried and then detected using XRD with Cu-Kα radiation at 40 kV and 30 mA (D8 Advance, Bruker, Germany). The samples were scanned from 3° to 65° 2θ with a scanning speed of 10°/min.

### ^1^H NMR and UV/vis Studies of Complexation of CIP with Naphthalene (Naph) in Solution

The ^1^H NMR spectra of CIP as π-acceptor were determined in methanol-*d*_4_ (Acros) in the mixture with a model π-donor compound (Naph) at room temperature on a Bruker-400 MHz NMR spectrometer (Bruker, Switzerland). The spectrometer was locked on the deuterium of the solvent, and the chemical shift (δ) was internally referenced to the proton of CIP. The UV/vis spectra of CIP–Naph mixtures in methanol/water solution (v:v, 1:1) at pH 2, 3, and 6 were acquired on a spectrometer (UV-1800, Shimadzu, Japan) against a solvent blank.

### Interaction of CIP with Adsorbents in Simulated Intestinal Fluids

Continuous interaction of CIP with GO, rGO, Mont and their mixtures was conducted in simulated intestinal fluids with bile salts after the adsorption of CIP in simulated gastric fluids in accordance with a previous method with modifications^55^. Desorption experiments were performed using the eight interaction systems to compare clearly the effects of Mont and pepsin. In brief, after the adsorption of CIP by the adsorbents in the gastric fluids, the solution (40 mL) was centrifuged to remove 30 mL supernatant and then amended by the same volume of the simulated intestinal fluid with 1000 mg/L bile salts to simulate intestinal digestion. All samples were shaken at 37 °C, and CIP concentration was determined at 1, 2, 3.5, and 4.5 h. The time at which intestinal fluid was amended was designated as 0 h.

### Data Analysis

The Freundlich models were used to fit the equilibrium adsorption isotherm data of CIP by GO, rGO and Mont at varying interaction systems (detailed in the SI). The *K*_d_ (L/g) value is the distribution adsorption coefficient calculated by dividing the adsorbed amount of the sorbate (*q*_e_) by its equilibrium concentration in aqueous phase (*C*_e_), *K*_d_ = *q*_e_/*C*_e_.

## Electronic supplementary material

Supplementary information
