# Supplementary material for: Interaction processes of ciprofloxacin with graphene oxide and reduced graphene oxide in the presence of montmorillonite in simulated gastrointestinal fluids
Source: Sci Rep. 2017 May 31;7:2588. doi: 10.1038/s41598-017-02620-4 (PMC5451422; doi:10.1038/s41598-017-02620-4)
Supplement: Supplementary file 1 — Supplementary information [file 41598_2017_2620_MOESM1_ESM.pdf]

Supplementary Information

Interaction processes of ciprofloxacin with graphene oxide and reduced graphene oxide in the presence of montmorillonite in simulated gastrointestinal fluids

Shuai Ma[1,a], Yang Si[2,a], Fei Wang[1,*], Lei Su[3], CongCong Xia[1], Jun Yao[1,4,**] , Huilun Chen[1], Xingyu Liu[5]

[1] School of Energy & Environmental Engineering, and Beijing Key Laboratory of Resource-oriented Treatment of Industrial Pollutants, University of Science and Technology Beijing, 30 Xueyuan Road, 100083 Beijing, China

[2] PPSM, CNRS UMR 8531, ENS-Cachan, 61 av President Wilson, 94230 Cachan, France

[3] Research Center for Bioengineering and Sensing Technology, School of Chemistry and Biological Engineering, University of Science and Technology Beijing, 100083, Beijing, China

[4] School of Water Resource and Environmental Engineering, Sino-Hungarian Joint Laboratory of Environmental Science and Health, China University of Geosciences (Beijing), 29 Xueyuan Road, Haidian District, 100083 Beijing, China

[5] National Engineering Laboratory of Biohydrometallurgy, General Research Institute for Nonferrous Metals, 100088, Beijing, China

---

Corresponding Author

* Corresponding author. Tel.: (86) 10 62333305; fax: (86) 10 62333305.

** Corresponding author. Tel.: (86) 10 82321958; fax: (86) 10 82321958.

E-mail addresses: wangfei@ustb.edu.cn (F. Wang), yaojun@ustb.edu.cn (J. Yao)

[a] S. Ma and Y. Si contributed equally to this work.

Characterization of GO, rGO and Mont. The surface functional groups on GO and rGO was obtained by using an AXIS Ultra DLD X-Ray photoelectron spectrometer (Kratos Analytical Ltd., UK) and Fourier transform infrared spectroscopy (FTIR) (NEXUS 670, Nicolet, USA). FTIR spectra were recorded in the 400-4000 cm^{-1} region with a resolution of 4 cm^{-1}. The surface area and porosity of GO and rGO were measured by using adsorption-desorption isotherms of N_2 at 77 K with a V-Sorb 2800P surface area and pore distribution analyzer (Gold APP Instruments Co., China). Raman spectra were obtained with a LabRamHRUV Raman spectrometer (Jobin-yvon HR800, FR), and the laser excitation was provided by an Ar^+ laser at a wavelength of 514 nm. The surface morphology of GO, rGO and Mont was determined by using a Nova NanoSEM 450 (FEI Ltd., Holland). And the surface morphologies of GO and rGO were also characterized through Field Emission Transmission Electron Microscope (Tecnai G2 F20, FEI Ltd., USA).

Data Analysis.

The Freundlich model was employed to fit the equilibrium adsorption data of CIP by GO, rGO and Mont.

Freundlich model:

$$q_e = K_{\mathrm{f}} \cdot C_e^{\,n}$$

where K_{f} is the adsorption coefficient ($(mg/g)/(mg/L)^n$), and n is a constant often used as an indicator of isotherm nonlinearity.

Table S1.

Selected properties of GO, rGO and Mont

	Purity (%) [a]	C(%)	O(%)	Thickness (nm)	out diameter [a] (nm)	layers[a]	SA [b] (m^2/g)	Pore volume[c] (cm^3/g)	Pore size [d] (nm)
rGO	>99	92.65	3.66	0.55~3.74	0.5-3	1-10	530	2.19	16.5
GO	>99	54.84	43.41	0.55~1.2	0.5~3	1-10	329	1.35	16.2
Mont	>99	-	-	-	-	-	66	0.15	9.5

[a] Provided by the supplier. [b] Surface area (SA) was measured by from the adsorption-desorption isotherm of N_2 at 77K by multipoint BET method. [c] pore volume was BJH adsorption cumulative volume with the pore diameter range of 2-150nm. [d] pore size was total adsorption average pore width (4V/A by BET, A is the surface area obtained by BET method).

Table S2.

Freundlich model fitting parameters for adsorption of CIP by GO, rGO and Mont at pH 2 under the varying interaction systems.

	K_f	n	r^2	K_d (C_e=2mg/L)
rGO	201±9	0.85±0.04	0.987	181
rGO+pepsin	60.9±2.8	0.80±0.02	0.995	53.0
rGO+Mont	781±31	0.50±0.03	0.968	552
rGO+Mont+pepsin	417±15	0.43±0.03	0.967	281
GO	84.1±5.5	0.79±0.05	0.981	72.7
GO+pepsin	27.5±2.3	0.93±0.04	0.988	26.2
GO+Mont	487±19	0.53±0.03	0.980	351
GO+Mont+pepsin	247±8	0.44±0.03	0.973	168
Mont	123±4	0.43±0.03	0.970	82.9
Mont+pepsin	115±3	0.43±0.02	0.979	77.5

K_f : (mg/g)/(mg/mL)^n. [b] Standard errors of K_f and n, respectively. K_d was derived from the equilibrium concentration of CIP at 2 mg/L.

Table S3.

Freundlich model fitting parameters for adsorption of pepsin by GO, rGO and Mont at pH 2

	K_f	n	r^2	K_d (C_e=300mg C/L)
rGO	96.88±19.43	0.88±0.04	0.989	48.86
GO	11.25±3.69	1.17±0.06	0.987	29.67
Mont	21.99±4.42	0.82±0.04	0.987	7.88

Table S4.

Dissolved Al and Si in varying interaction systems containing Mont.

	Al (mg/L)	Si (mg/L)
Mont+CIP	1.20	5.42
Mont+pepsin+CIP	0.99	5.02
Mont+rGO+CIP	1.57	7.79
Mont+rGO+pepsin+CIP	1.38	6.34
Mont+GO+CIP	1.67	7.93
Mont+GO+pepsin+CIP	1.46	6.99

Table S5.

Freundlich model fitting parameters for adsorption of CIP by GO, rGO and Mont at pH 6.5

	K_f	n	r^2	K_d (C_e=2mg/L)
rGO	134±9	0.73±0.04	0.982	111
rGO+Mont	356±16	0.46±0.03	0.948	245
GO	47.6±7.1	1.15±0.11	0.964	52.82
GO+Mont	253±9	0.77±0.04	0.984	215
Mont	28.28±1.91	0.95±0.05	0.983	27.32

K_f : (mg/g)/(mg/mL)^n. [b] Standard errors of K_f and n, respectively. K_d was derived from the equilibrium concentration of CIP at 2 mg/L.

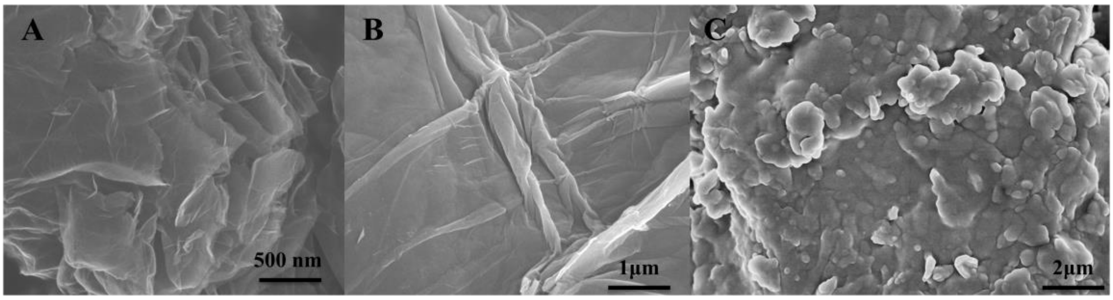

Fig. S1. SEM images of rGO (A), GO (B), and Mont (C).

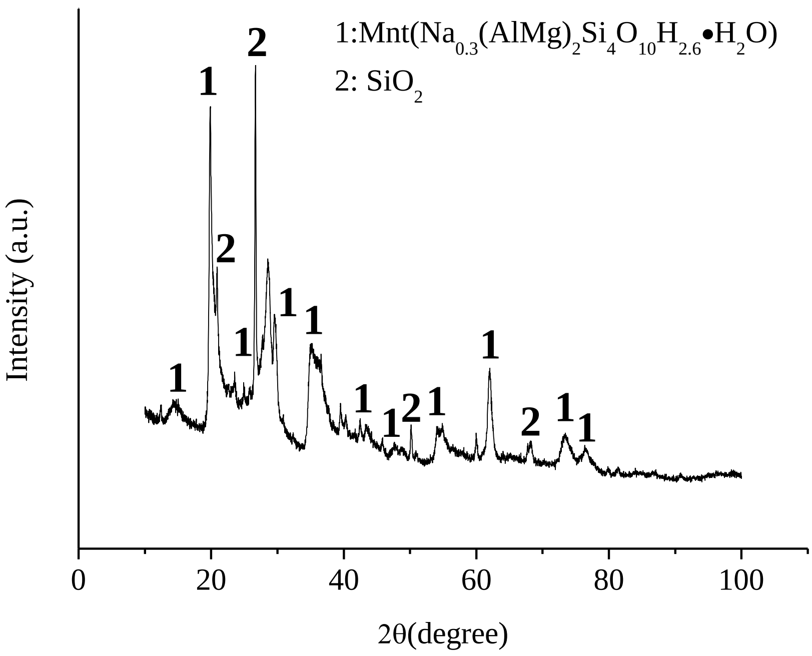

Fig. S2. XRD image of Mont.

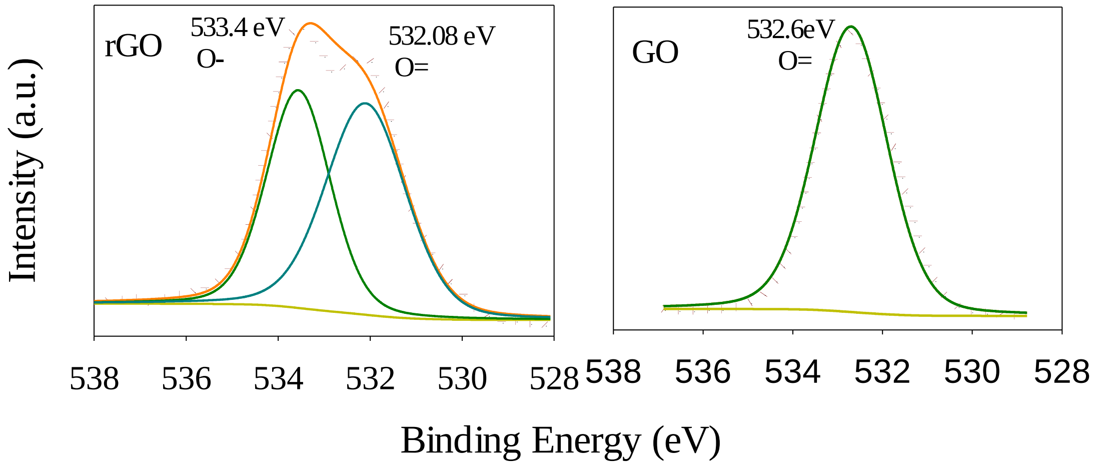

Fig. S3. XPS spectra of rGO and GO for O 1s.

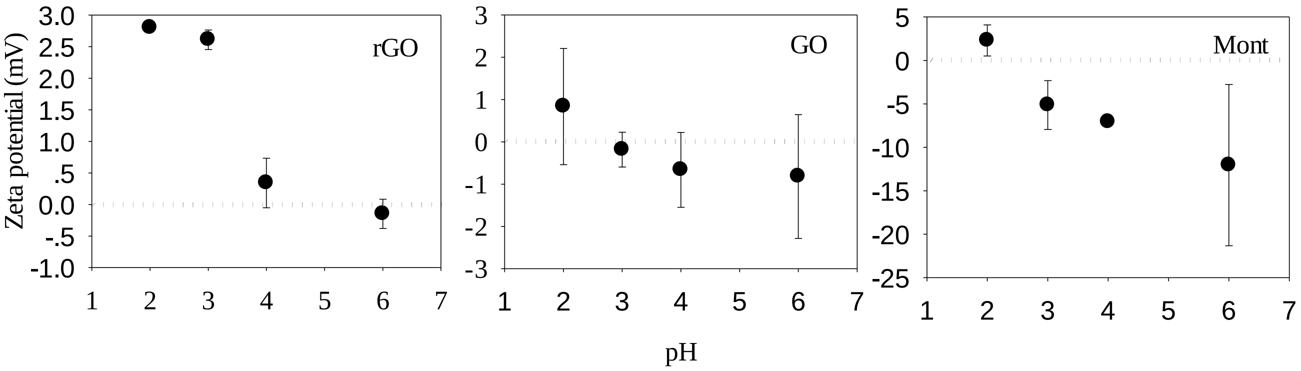

Fig. S4. Zeta potential curves versus pH of rGO, GO and Mont.

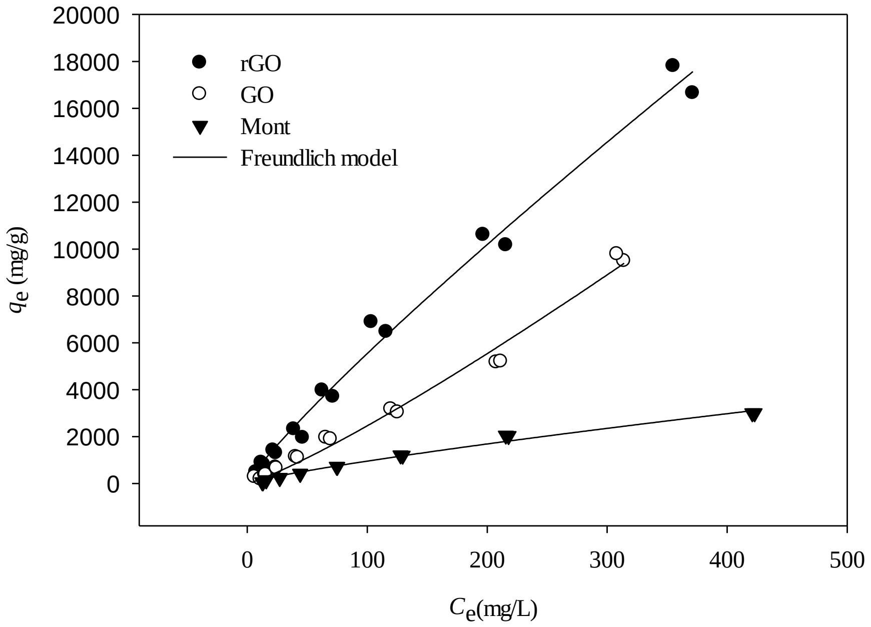

Fig. S5. Adsorption isotherms of pepsin onto rGO and GO. Pepsin concentration was determined by TOC. The q_e (mg/g) is the solid-phase concentration and C_e (mg/L) is the equilibrium concentration.

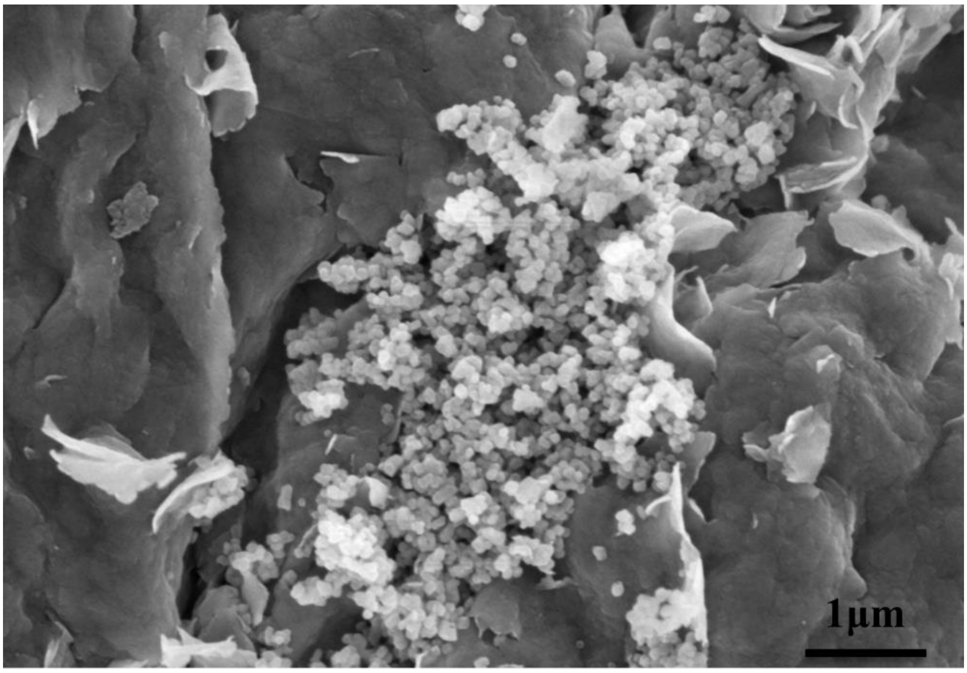

Fig. S6. SEM image of rGO+Mont+pepsin+CIP at pH 2.

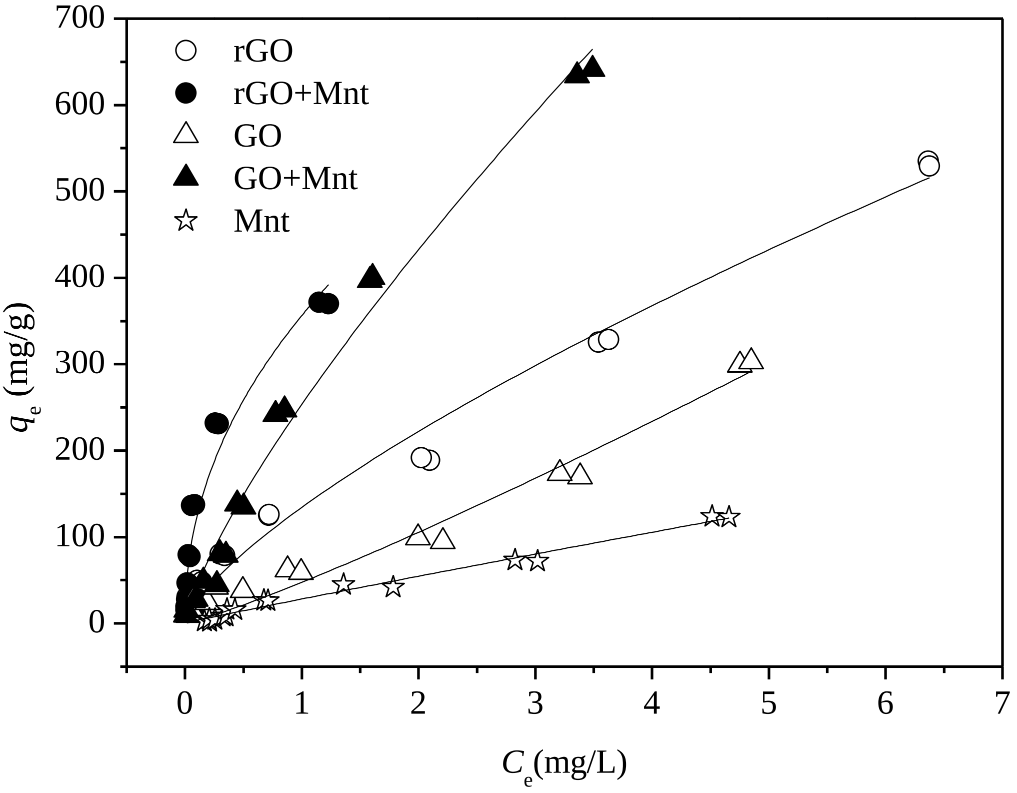

Fig. S7. Adsorption of CIP on GO, rGO and Mont under the varying interaction system at pH 6.5. The solid line is Freundlich model.

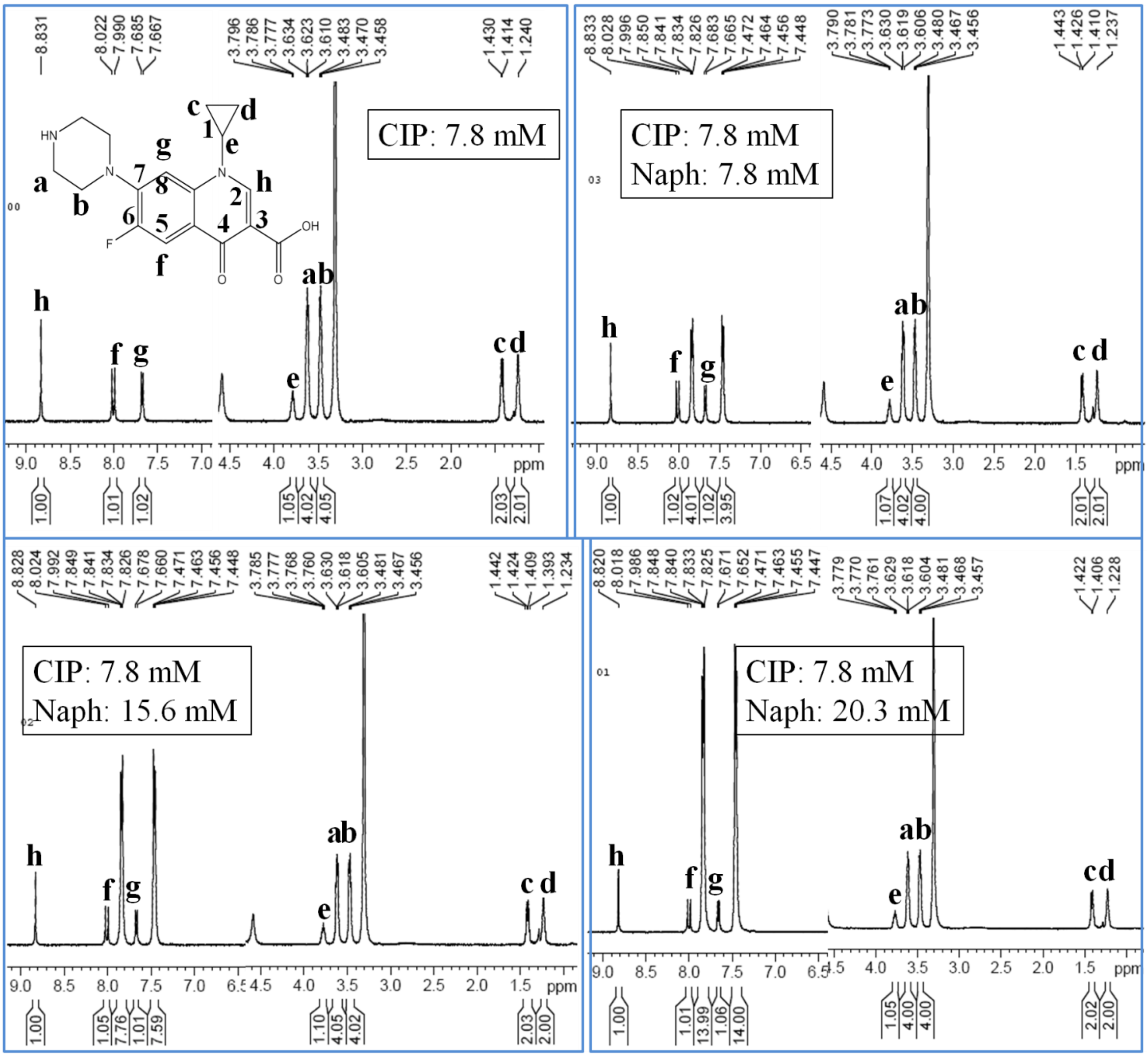

Fig. S8. ^{1}H NMR spectra of CIP as π-acceptors in methanol-d_4 affected by complexation with Naph as π-donors. CIP concentration was 7.8 mM, and Naph concentration was 0, 7.8, 15.6 and 20.3 mM. a, b, c, d, e, f, g, and h were noted as H position in CIP molecular.

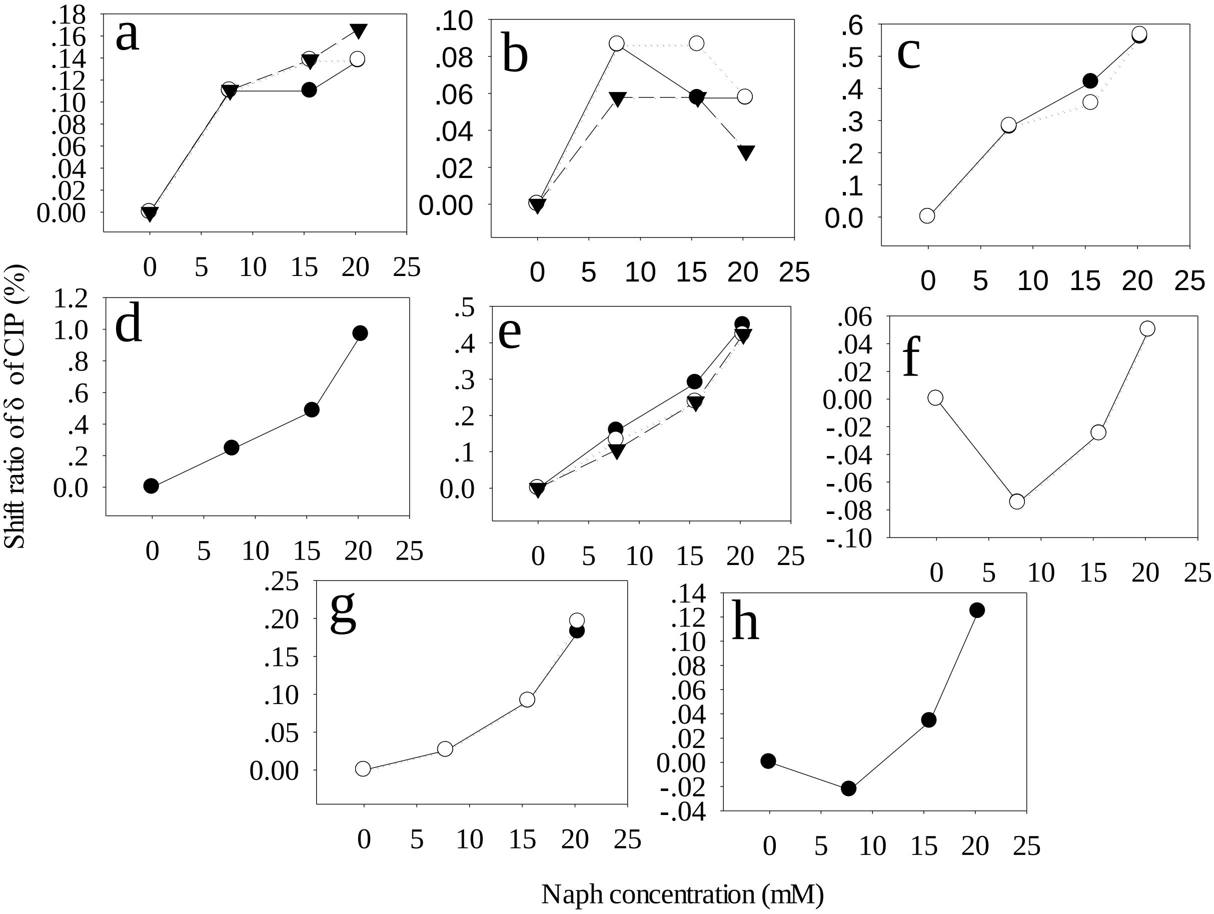

Fig. S9. ^{1}H NMR chemical shift ratio of CIP as π-acceptors in methanol-d_4 affected by complexation with Naph as π-donors. CIP concentration was 7.8 mM, and Naph concentration was 0, 7.8, 15.6 and 20.3 mM. a, b, c, d, e, f, g, and h were noted as H position in CIP molecular, as showed in Figure 4.

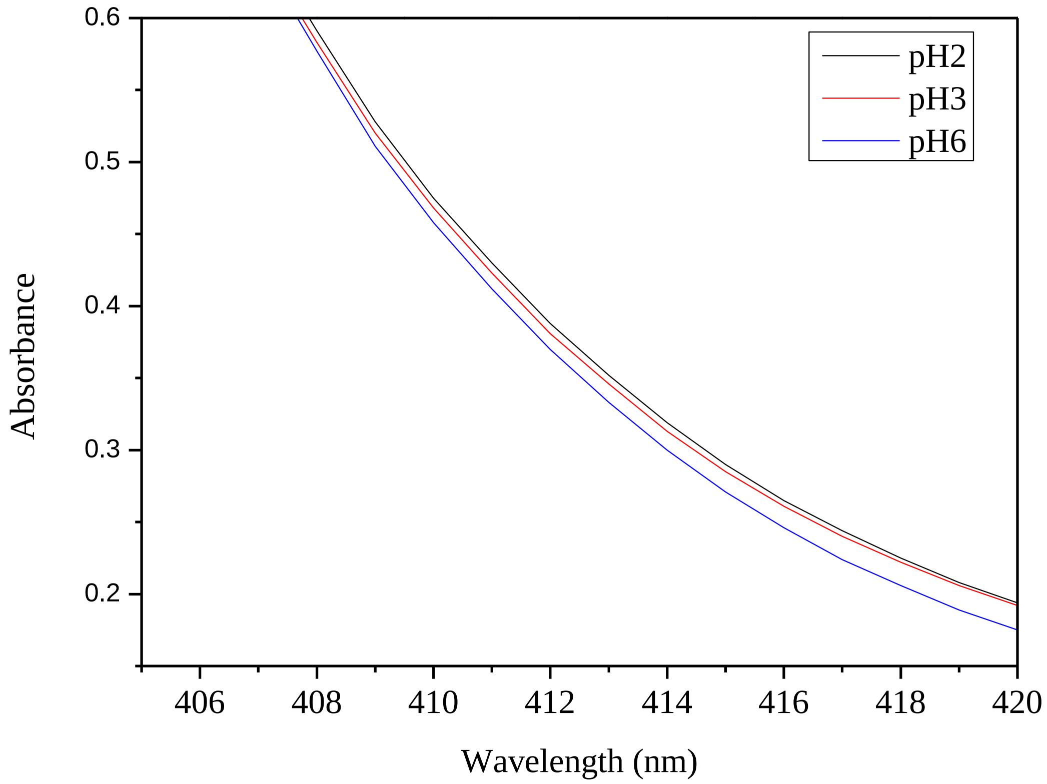

Fig. S10. UV/vis difference spectra in mixed solution of water and methanol (v/v, 1:1) showing the charge-transfer absorption band of π-π complexes between CIP (0.02 M) and Naph (0.02 mM) and its dependence on the solution pH.

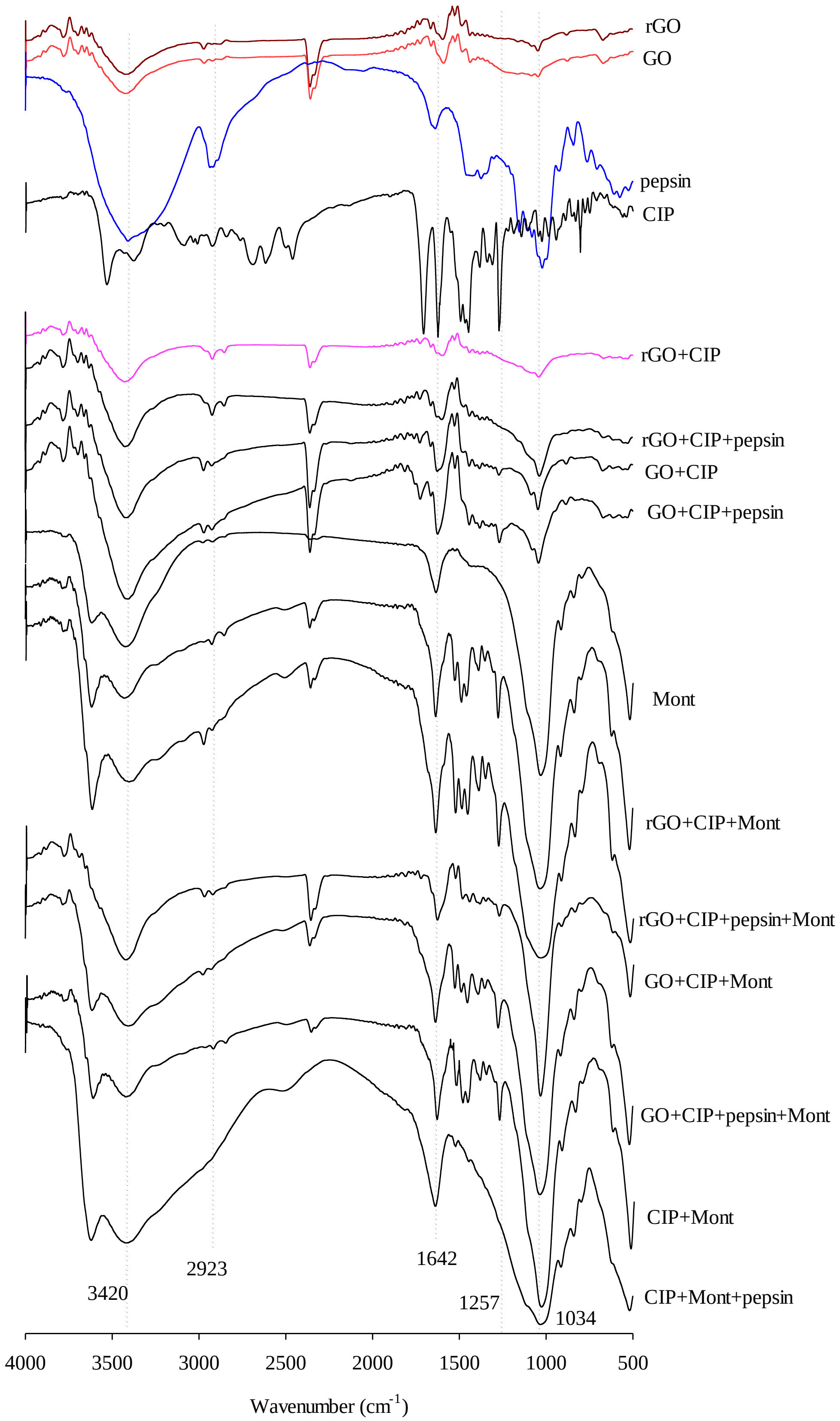

Fig. S11. FTIR spectra of GO, rGO and Mont under the varying interaction systems after sorption at the range of 400 to 4000 cm^{-1} at pH 2.

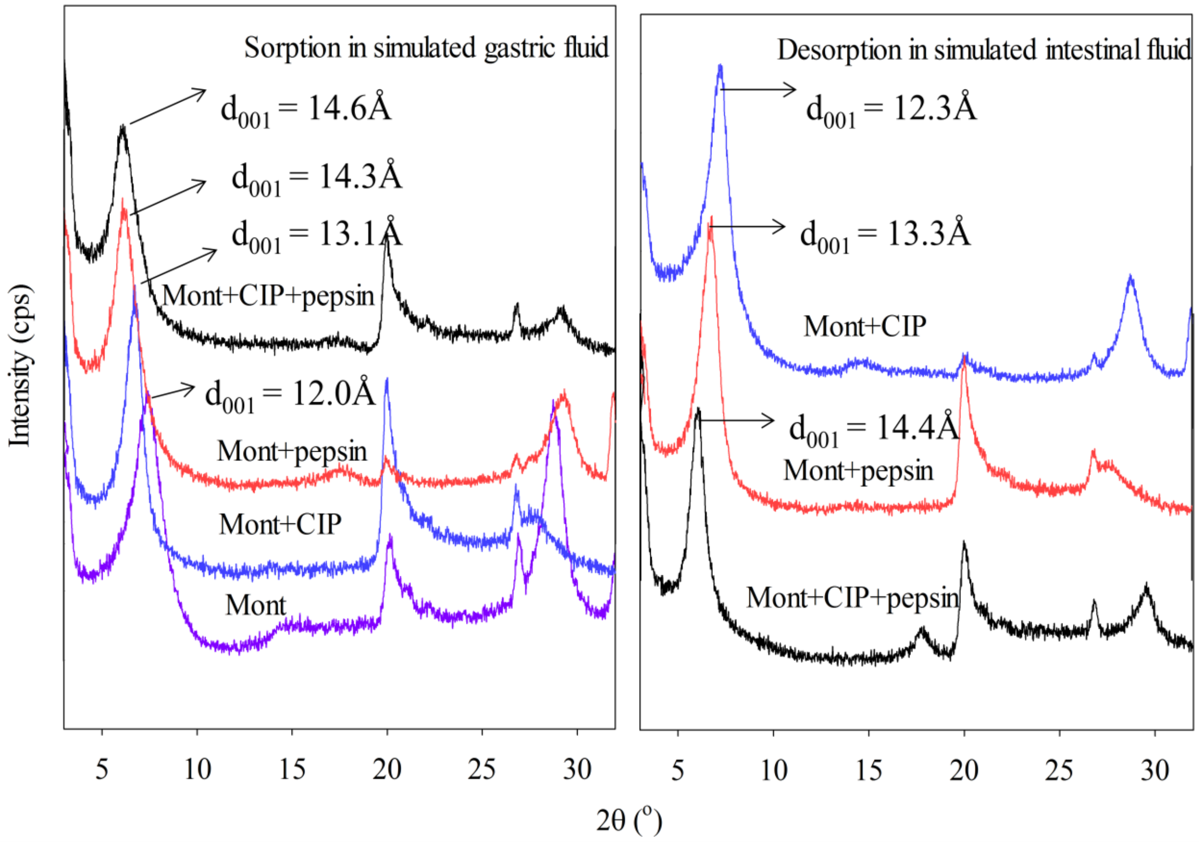

Fig. S12. XRD patterns of Mont after sorption of CIP, pepsin and CIP+pepsin in simulated gastric fluid, and then desorption in simulated intestinal fluid.
